# Everybody's Page

**Published:** 1892-10-29

**Authors:** 


### EVERYBODY'S PAGE.

THE extent to which the sale of cigar ends is carried on has attracted the eagle eye of the Inland Revenue Department, which is anxious to prove that to pick up cigar ends and to sell them is to sell uncustomed tobacco. A great business is carried on in the East End in these other ends, which, it is alleged, go to fill the carcase of the British cigar. It is satisfactory to know that the East End cigar contains nothing more harmful than the ends of cigars, which have been cut off in music halls and restaurants.

THOSE who are in search of mild yet bracing air for the winter need not go beyond these shores, though it is the fashion for those with means to turn their back on Great Britain as soon as the first signs of winter approach. Among numerous other places where plenty of sunshine and a practical immunity from snow and long frost are to be found is Tenby, which has been described as possessing a summer temperature of the South of Norway, and a winter temperature of the Mediterranean.

THE evidence of Mr. Thomas Hawksley, the engineer, before the Royal Commission on the London Water Supply, produced some information both reassuring and to many people novel. Cholera, he asserts, always goes up rivers, and not down them, which is a very remarkable fact. Even if this were not the case, the fear of infection through rivers is not as great as is generally supposed, owing to the purifying process of oxidation which is always at work in running streams, a process which cannot take place in wells which are highly polluted.

Is anything to be gained by taxing a horse beyond endurance? The military ride between Vienna and Berlin has at least shown one thing, that men of education can be guilty of as gross cruelty as the most ignorant costermonger. It is admitted that the powers of many of the horses were taxed beyond all endurance, and nothing can be gained by continuing to force an animal after it has clearly shown it has done its willing best. The riders have been treated as heroes. Should not the Austrian Society for the Prevention of Cruelty to Animals have stepped in and stopped an exhibition which is neither flattering to the hearts nor heads of people supposed to be civilised?

"ROSE-COLD" is the name by which hay fever is known in the United States, owing to the fact that roses give rise in some people to a very severe form of hay fever. Though heat alone cannot cause the fever, the sun's rays are sufficient, as many people have experienced, to cause sneezing owing to irritation set up. As the affection is one from which people of nervous temperament suffer, it chiefly devotes its attention to the educated classes, more especially those dwelling in towns.

TOBACCO is becoming quite popular in the medical world. On the one hand we hear smoking recommended as a safeguard against cholera, and on the other hand we are told by a medical man that his experience goes to prove that tobacco smoke, as inhaled by the habitual smoker, retards or prevents the development of the bacillus of tuberculosis in the larynx and lungs of the smoker. The same doctor states that during a practice of 20 years he does not remember one case of consumption in a habitual smoker.

THERE are, doubtless, many solid objections to the exclusive management by the State of public houses and of the drink traffic; but State management would have one distinct advantage, in that the motive to sell adulterated liquors being removed the public would be protected against the poisonous fluids which are abroad now. The effect of this on temperance is not so easy to gauge. The knowledge that all alcoholic drinks were good and pure might prove a direct incentive to some thirsty people to drink even more than had previously been their habit. The question truly bristles with difficulties.

IN Kidderminster they object to the importation of Eastern carpets, of course, purely on public spirited grounds and not from any sordid considerations of self interest. Carpets of all kinds are known to possess unlimited capabilities of harbouring dust, and no doubt every germ under the sun. Eastern carpets, therefore, in these times of cholera, are possibly conveyers of contagion, but not many people would go so far as one enthusiastic resident in Kidderminster, who would prefer to have a tigress roaming unchained about his house to having an Eastern carpet in the town!

